# Analysis of Light- and Carbon-Specific Transcriptomes Implicates a Class of G-Protein-Coupled Receptors in Cellulose Sensing

**DOI:** 10.1128/mSphere.00089-17

**Published:** 2017-05-10

**Authors:** Eva Stappler, Christoph Dattenböck, Doris Tisch, Monika Schmoll

**Affiliations:** a Center for Health and Bioresources, AIT Austrian Institute of Technology GmbH, Tulln, Austria; b TU Wien, Insitute of Chemical Engineering, Research Area Molecular Biotechnology, Vienna, Austria; Carnegie Mellon University

**Keywords:** CAZyme clusters, *Hypocrea jecorina*, *Trichoderma reesei*, cellulase gene expression, heterotrimeric G-protein pathway, light response, signal transduction, surface sensing

## Abstract

In fungi, most metabolic processes are subject to regulation by light. *Trichoderma reesei* is adapted to degradation of plant cell walls and regulates production of the required enzymes in a manner dependent on the nutrient source and the light status. Here we investigated the interrelated relevance of two regulation levels of the transcriptome of *T. reesei*: light regulation and carbon source-dependent control. We show that the carbon source (cellulose, lactose, sophorose, glucose, or glycerol) is the major source of variation, with light having a modulating effect on transcript regulation. A total of 907 genes were regulated under cellulase-inducing conditions in light, and 947 genes were regulated in darkness, with 530 genes overlapping (1,324 in total). Only 218 of the 1,324 induction-specific genes were independent of light and not regulated by the BLR1, BLR2, and ENV1 photoreceptors. Analysis of the genomic distribution of genes regulated by light upon growth on cellulose revealed considerable overlap of light-regulated clusters with induction-specific clusters and carbohydrate-active enzyme (CAZyme) clusters. Further, we found evidence for the operation of a sensing mechanism for solid cellulosic substrates, with regulation of genes such as *swo1*, *cip1*, and *cip2* or of genes encoding hydrophobins which is related to the cyclic AMP (cAMP)-dependent regulatory output of ENV1. We identified class XIII G-protein-coupled receptors (GPCRs) CSG1 and CSG2 in *T. reesei* as putative cellulose/glucose-sensing GPCRs. Our data indicate that the cellulase regulation pathway is bipartite, comprising a section corresponding to transcriptional regulation and one corresponding to posttranscriptional regulation, with the two connected by the function of CSG1.

**IMPORTANCE** In fungi, most metabolic processes are subject to regulation by light. For *Trichoderma reesei*, light-dependent regulation of cellulase gene expression is specifically shown. Therefore, we intended to unravel the relationship between regulation of enzymes by the carbon source and regulation of enzymes by light. Our two-dimensional analysis included inducing and repressing carbon sources which we used to compare light-specific regulation to dark-specific regulation and to rule out effects specific for a single carbon source. We found close connections with respect to gene regulation as well as significant differences in dealing with carbon in the environment in light and darkness. Moreover, our analyses showed an intricate regulation mechanism for substrate degradation potentially involving surface sensing and provide a basis for knowledge-based screening for strain improvement.

## Abstract (precis)

In fungi, most metabolic processes are subject to regulation by light. For *Trichoderma reesei*, light-dependent regulation of cellulase gene expression is specifically shown. Therefore, we intended to unravel the relationship between regulation of enzymes by the carbon source and regulation of enzymes by light. Our two-dimensional analysis included inducing and repressing carbon sources which we used to compare light-specific regulation to dark-specific regulation and to rule out effects specific for a single carbon source. We found close connections with respect to gene regulation as well as significant differences in dealing with carbon in the environment in light and darkness. Moreover, our analyses showed an intricate regulation mechanism for substrate degradation potentially involving surface sensing and provide a basis for knowledge-based screening for strain improvement.

## INTRODUCTION

In nature, efficient degradation of the substrate at hand and optimal distribution of resources to support this process are of crucial importance for survival of and successful competition by a fungus. *Trichoderma reesei* (syn. *Hypocrea jecorina*) is one of the most potent plant biomass degraders applied in industry (1, 2) and has become a model system for this task (3, 4). Production of enzymes needed for degradation of a given substrate is adjusted to its chemical structure and to the light status, pH, and other environmental factors (5). Therefore, signal transduction pathways represent important targets for strain improvement (6, 7).

Cellulase genes (8, 9) as well as numerous glycoside hydrolase genes are regulated by light in *T. reesei* (10), and photoreceptors were shown to regulate cellulase gene expression in *T. reesei* (11) and in *Neurospora crassa* (12). Consequently, crosstalk of gene regulation in response to light and nutrient signal transduction with plant cell wall degradation as the output pathway has gained increased attention in recent years (6, 13). Here, the heterotrimeric G-protein pathway (14) in particular, as well as the downstream cyclic AMP (cAMP) pathway, was found to be important and to impact cellulase gene expression in a light-dependent manner. Nevertheless, constitutive activation of G-alpha subunits was not sufficient to achieve inducer-independent cellulase production (10, 15–18). The function of G-protein alpha subunits GNA1 and GNA3 in cellulase gene expression indicates that the upstream G-protein-coupled receptors (GPCRs) are also relevant for cellulase regulation. *T. reesei* has 57 predicted GPCRs (1), but only the pheromone receptors have been characterized thus far (19). Interestingly, the genomic locus of the homologue of *N. crassa* carbon sensor GPR-4 (20) is not present in the genome of *T. reesei* (21). Nevertheless, a recent study in *N. crassa* revealed several other GPCRs with phenotypes on cellulose (22); hence, cellulose sensing also by the heterotrimeric G-protein pathway in *T. reesei* would not be without precedent.

Cellulase gene expression occurs on different carbon sources, which in most cases reflect the presence of individual degradation products of plant cell walls (23–25). However, when an easily metabolizable carbon source such as glucose is detected, the production of plant cell wall-degrading enzymes is repressed by a mechanism called carbon catabolite repression in order to avoid wasting of resources (26). The presence of the strongly cellulase-inducing polysaccharide cellulose most closely resembles the conditions *T. reesei* encounters in nature. The most complete set of plant cell wall-degrading enzymes is secreted on this carbon source (27, 28). However, the presence of sophorose, which is a transglycosylation product derived from cellobiose and is considered the natural inducer, also leads to strong induction even when present in minute amounts at a concentration of only 1.5 mM (29). Hence, growth in the presence of sophorose specifically reveals mechanisms related to early induction. The starvation response is likely to play a role in cellulase induction in fungi (3), which is assumed to contribute to the response to sophorose in *T. reesei* (30). However, transfer of cells to medium lacking a carbon source does not induce cellulase genes in a manner similar to that seen in the presence of sophorose (31).

*T. reesei* is one of only few fungi that produce cellulases on lactose, which is assumed to be associated with a degradation pathway of hemicelluloses (32). The precise mechanism of this induction is subject to ongoing research (33, 34). In addition to the results seen with cellulases, the pentose phosphate pathway needed to degrade lactose, for example, was also found to be subject to regulation by light and photoreceptors (11). Glucose represses genes encoding plant cell wall-degrading enzymes (due to carbon catabolite repression), whereas upon growth on glycerol as the carbon source, *T. reesei* does not produce cellulases, but addition of an inducer—for example, sophorose—still leads to cellulase induction (30), which is not the case for glucose.

Changing light conditions, caused by the rotation of earth resulting in day and night or by growth on the surface of or within a substrate, result in considerably altered physiological processes and metabolic responses in fungi (35, 36). Light-dependent processes in *T. reesei* include development, cellulase gene expression (and expression of glycoside hydrolases in general), carbon utilization, and sulfur metabolism (37, 38). Regulation of several of these processes in response to light in *T. reesei* is achieved by the BLR1, BLR2 and ENV1 photoreceptors, by the heterotrimeric G-protein pathway, by the cAMP pathway, and, presumably, by the sulfur controller LIM1 (6, 10, 13). With respect to the G-protein pathway, light-dependent positive effects on cellulase gene expression were shown for G-alpha subunits GNA1 (17) and GNA3 (15) and also for beta and gamma subunits GNB1 and GNG1 (10). These studies indicate a strong regulatory interrelationship between nutrient signaling and light response, in which ENV1 plays a major role in connecting the two signaling cascades (11, 18). The BLR1 and BLR2 photoreceptors represent GATA-type transcription factors which exert their function as a complex (9, 13). However, individual functions of photoreceptors have also been observed in *T. reesei* and *N. crassa* (11, 12). The ENV1 photoreceptor is assumed to modulate the function of BLR1 and BLR2 by interaction, although individual regulatory targets are also known for ENV1 (11, 39). Interestingly, BLR1, BLR2, and ENV1 also have regulatory (direct and indirect) targets in darkness (11, 39), and residual light-dependent regulation in their absence (9, 11) indicates operation of additional light-dependent mechanisms.

In this report, we provide a view on the data from different angles and evaluate regulation by light and photoreceptors as well as regulation in response to inducing, noninducing, or repressing conditions. We connect induction-specific gene expression to the functions of photoreceptors and evaluate prioritization of signals and genomic distribution of regulated genes to relate them to carbohydrate-active enzyme (CAZyme) clusters. We then compare the data sets either according to the effect of the carbon sources used (inducing/noninducing or repressing) or according to effect of the light conditions used (constant light or constant darkness). This two-dimensional analysis enables us to evaluate distinct regulation patterns in light and darkness and to discern induction-specific regulation that is independent of light.

## RESULTS

### Light-dependent gene regulation across different carbon sources.

**FIG 1:**
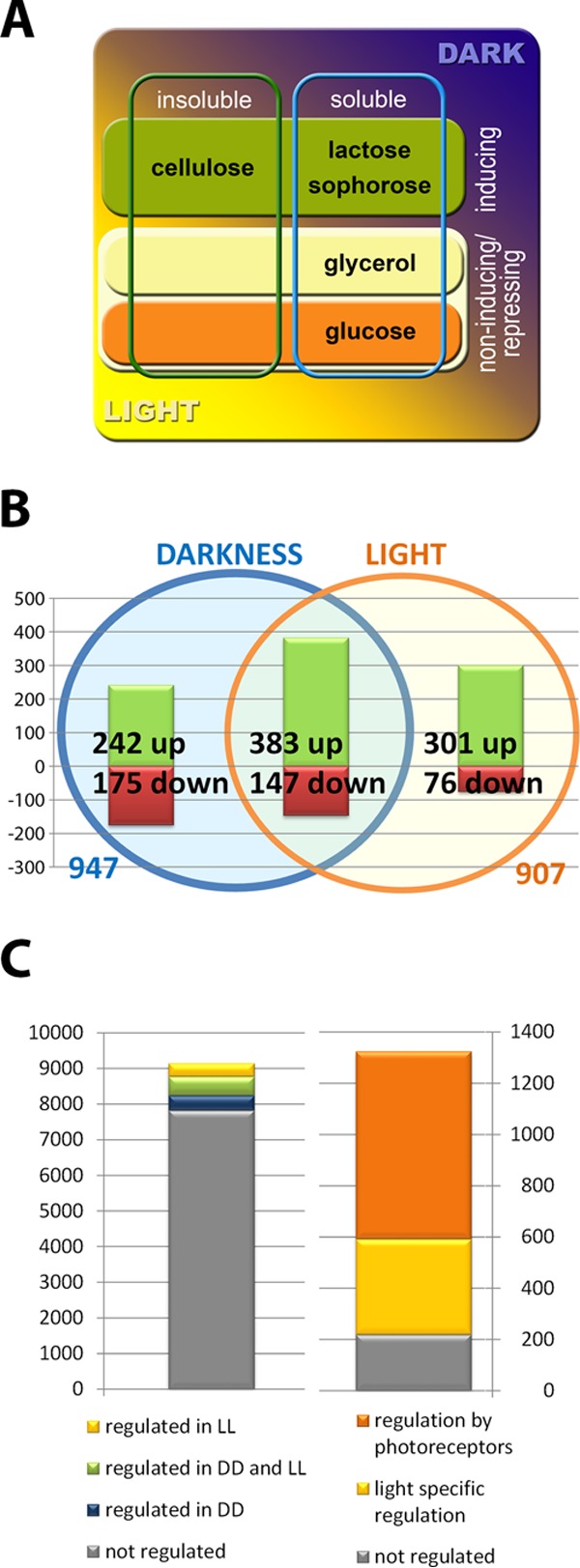
Experimental approach and light-specific regulation. (A) *T. reesei* was cultivated on the carbon sources cellulose, lactose, sophorose, glycerol, and glucose for evaluation of nutrient-dependent gene regulation (first dimension). All carbon sources were used for cultivation in constant light and in constant darkness (second dimension) to distinguish light-dependent effects. Cellulose is an insoluble, inducing carbon source, whereas lactose and sophorose are inducing but soluble. Glycerol is noninducing and soluble, and glucose is a repressing and soluble carbon source. (B) Gene regulation specific to cellulase-inducing conditions. Genes were upregulated (green) or downregulated (red) at least 2-fold under cellulase-inducing conditions in constant light or in constant darkness or under both conditions (*P* value = 0.01 [FDR]). (C) Genes regulated under induction-specific conditions, by light or by photoreceptors. For regulation by photoreceptors, data were taken from the data set published in reference 11, which describes gene regulation by the photoreceptors BLR1, BLR2, and ENV1 in light and darkness upon growth on cellulose. LL, constant light; DD, constant darkness.

In order to gain insight into carbon source-specific gene regulation in *T. reesei*, we performed transcriptome analysis of cultures grown on the cellulase-inducing carbon sources cellulose, lactose, and sophorose as well as on the repressing carbon source glucose and on the noninducing carbon source glycerol. Since it was shown that light has a profound influence on gene regulation in *T. reesei*, including regulation of glycoside hydrolases, we additionally used constant light and constant darkness for our cultivations (Fig. 1A). This experimental design enabled us to evaluate carbon source-specific effects and whether these effects are dependent on the light status.

We analyzed genes that are differentially regulated between light and darkness on glucose, glycerol, cellulose, lactose, and sophorose. Therefore, all samples derived from cultivations in light were treated as replicates versus all samples derived from cultivations in dark. Those genes are assumed to be relevant for the general reaction of *T. reesei* to light (light sensitivity). We found 206 genes with significantly different regulation results; 100 showed a positive response to light, while 106 reacted to light with decreased transcript levels (see Data Set S1 in the supplemental material).

10.1128/mSphere.00089-17.2 DATA SET S1  Carbon-independent gene regulation by light. Download DATA SET S1, XLS file, 0.2 MB. Copyright © 2017 Stappler et al. 2017 Stappler et al. This content is distributed under the terms of the Creative Commons Attribution 4.0 International license.

As expected, *env1*, the photolyase gene *phr1*, *frq1*, and the homologue to the *N. crassa* light-responsive transcription factor *sah-1*, all of which are known to be regulated in a light-dependent manner (8, 11, 40–42), were found among the genes upregulated in light and hence confirmed the validity of our study. Additionally, seven genes associated with sexual development, including *hpp1*, *hpr2*, *mat1-2-1*, and genes encoding several CAAX processing enzymes, were in this gene set, which is in accordance with earlier data (11) and with the preference of *T. reesei* for sexual development in light along with rather low stringency with respect to carbon requirements (43). However, *lxr1*, encoding l-xylose reductase, as well as genes encoding two putative alpha glycosidases (TR_65333 and TR_55802), one putative beta glycosidase (TR_39942), the chitinase *chi18-9*, and a candidate trehalase (TR_123226) were also positively regulated by light. Genes downregulated in light included those encoding two family 16 glycoside hydrolases (beta-glycosidases; TR_65406 and TR_48274) and hydrophobin genes *hfb2* and *hfb3* as well as genes encoding four predicted G-protein-coupled receptors (TR_72004/class V, TR_37525/class VI, TR_124113/PTH11-like, and TR_76763/PTH11-like).

### Gene regulation under cellulase-inducing conditions in light and darkness.

Both light and photoreceptors have a strong influence on metabolism and transcription of glycoside hydrolases (10, 11). Consequently, we wanted to investigate in more detail whether induction-specific gene regulation across different inducing carbon sources is modulated in light and darkness. Therefore, we separated the data sets obtained from cultures grown in light from those obtained from cultures grown in darkness. Samples from cultures grown under inducing conditions (cellulose, lactose, sophorose) were treated as replicates versus those from cultures grown under noninducing/repressing conditions (glucose, glycerol).

Our first level of analysis was identification of induction-specific genes, which were the focus of our study. We did this analysis separately in light and darkness and found that induction-specific regulation does occur in light and darkness but that the number of genes with induction-specific regulation only in darkness or only in light is considerable (Fig. 1B). In the following, we discuss the genes regulated in an induction-specific manner only in light, those regulated in an induction-specific manner only in darkness, and those regulated in an induction-specific manner specifically in light and darkness along with characteristics of their genomic localizations.

We found 907 genes regulated under inducing conditions in light and 947 genes regulated under inducing conditions in darkness. Of those, 530 genes (383 upregulated, 147 downregulated; Fig. 1B) were regulated specifically under inducing conditions in light and darkness (Data Set S2). We found 377 genes that were regulated specifically under inducing conditions in light, while 417 genes were regulated specifically under inducing conditions in darkness (242 upregulated, 175 downregulated) (Fig. 1B). Interestingly, among the 1,324 genes specifically regulated under inducing conditions in light and/or darkness (induction specific, light sensitive, or insensitive), we found several homologues of the genes proposed to be involved in carbon scouting (44) to be upregulated. Of those, endoglucanase genes *cel7b* (*egl1*) and *cel5a* (*egl2*), the β-xylosidase *bxl1* gene, the arabinofuranosidase *abf3* gene, the β mannosidase TR_62166 gene, and the xylose reductase TR_107776 gene are upregulated in light and darkness, while TR_112134 and TR_3481 as well as the GH115 encoding TR_79606 are genes whose induction is specifically upregulated only in light.

10.1128/mSphere.00089-17.3 DATA SET S2  Induction-specific gene expression in light and darkness and regulation by photoreceptors. Download DATA SET S2, XLS file, 1.2 MB. Copyright © 2017 Stappler et al. 2017 Stappler et al. This content is distributed under the terms of the Creative Commons Attribution 4.0 International license.

In order to get a first overview of the relevance of induction-specific regulation to metabolic pathways, we used the metabolic model of *T. reesei* provided in reference 45. In this model, functions in primary metabolism, including the tricarboxylic acid cycle and glycolysis, are assigned to *T. reesei* gene models. Of the 1,324 induction-specific genes that we had found, 109 are among the genes in this metabolic model (Data Set S3). Part of the pentose phosphate pathway had been shown previously to be subject to regulation by light and photoreceptors (11). With respect to glycolysis, we found several genes (*tpi1a*, *dak2-2c*, *gut1*, *gld1*) to be downregulated in one or more photoreceptor mutants (11) in light in the pathway leading to glycerol. *gut1* and *dak2-2c* are specifically induction regulated as well. Interestingly, except for *gld1*, these genes also show a positive correlation with the specific protein production rate (SPPR) in growth rate-controlled continuous fermentations (45). In contrast, *eno1-2*, *pyk1-2*, and *pcd1-5-6*, which encode proteins acting subsequently in the glycolysis pathway from 3-phospho-d-glycerate to acetaldehyde, are negatively correlated with the specific protein production rate. These genes are all positively regulated by ENV1 in light, but the regulation does not take place in an induction-specific manner. Also, the gene coding for the subsequently acting ACS1-2a is strongly downregulated upon deletion of *env1*. In the glyoxylate cycle, isocitrate lyase gene *icl1* is induction specific and is positively regulated by BLR1 and ENV1 in light. *cit1* and *cit2*, genes encoding citrate synthase and 2-methylcitrate synthase, are regulated by ENV1 as well and are negatively correlated with the SPPR. Consequently, the central pathways of primary metabolism, in particular, the pentose phosphate pathway and the glycolysis pathway, are direct and/or indirect targets of light responses. Interestingly, we found an overlap of negative correlation of the SPPR (45) with positive regulation by ENV1 in light in several cases (Data Set S3).

10.1128/mSphere.00089-17.4 DATA SET S3  Induction-specific regulation of metabolic pathways as well as regulation of the genes of the metabolic model by photoreceptors. Download DATA SET S3, XLSX file, 0.2 MB. Copyright © 2017 Stappler et al. 2017 Stappler et al. This content is distributed under the terms of the Creative Commons Attribution 4.0 International license.

### Regulation of genes in CAZyme clusters under inducing conditions.

We were interested in how many CAZyme-encoding genes were among those we found to be regulated under inducing conditions. Therefore, we repeated the analysis done by Martinez et al. (46), which is shown only partially in the supplementary materials there, applying a revised and updated annotation of CAZyme genes (1). We adjusted the stringency to obtain roughly the same clusters as those obtained with the analysis reported earlier (46) and checked them for potential misassembly using the high-quality assembly method described in reference 47. We then found 39 CAZyme clusters instead of only 25. Of the 189 genes in these clusters, 37 overlapped with our induction-specific genes. Twenty-three of them, including *cbh1* (*cel7a*), *cbh2* (*cel6a*), *egl1*, *egl2*, *egl4*, *xyn2*, and *xyn4*, were upregulated in light and darkness (Data Set S4), with the majority of these genes positively regulated by BLR1 and ENV1 in light (11). Interestingly, however, there was no CAZyme cluster that consisted entirely of genes that were coregulated in response to inducing conditions or to light. Four genes, including *bgl1* and *egl3*, were found to be upregulated only under inducing conditions in darkness, while seven genes, including the beta glucosidase gene *cel3e* and the predicted trehalase gene TR_123226, were upregulated only in light. Only three of the CAZyme cluster genes were found to be downregulated under inducing conditions: the chitinase *chit18-18* gene in light and TR_122511 and TR_111897 in darkness. We then evaluated clustered regulation of induction-specific genes. Of the 1,324 genes in total that were found to be regulated under inducing conditions, 295 were found to be nonrandomly distributed within the genome and are assigned to 28 clusters (Data Set S4). Overlaps were found only for cluster 5 (scaffold 3, 432000 to 518000), cluster 14 (scaffold 7, 1063000 to 1232000), cluster 22 (scaffold 18, 116000 to 199000), and cluster 23 (scaffold 19, 41000 to 281000), with (in total) 6 CAZyme-encoding genes present among the 37 induction-specific ones found in CAZyme clusters.

10.1128/mSphere.00089-17.5 DATA SET S4  Genomic clusters associated with CAZyme genes, induction, or gene expression on cellulose. Download DATA SET S4, XLSX file, 0.3 MB. Copyright © 2017 Stappler et al. 2017 Stappler et al. This content is distributed under the terms of the Creative Commons Attribution 4.0 International license.

### Nonrandom genomic distribution of genes regulated by light on cellulose.

Evaluation of the genomic distribution of genes regulated by light on different carbon sources revealed 15 clusters for cellulose (containing 66 genes; Data Set S4) but only 1 for sophorose (11 genes), 2 for glucose (14 genes), and none for the other carbon sources.

**FIG 2:**
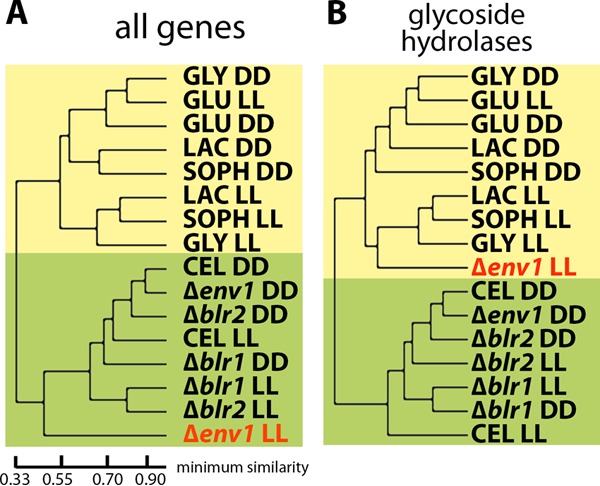
Clustering of genome-wide regulation patterns. Hierarchical cluster analysis of (A) all genes or (B) glycoside hydrolases of the wild-type strain grown on cellulose (CEL), lactose (LAC), sophorose (SOPH), glycerol (GLY), and glucose (GLU) and the Δ*blr1*, Δ*blr2*, and Δ*env1* mutants grown on cellulose (11) in constant light (LL) and constant darkness (DD) was performed. Data used for hierarchical cluster analysis by HCE3.5 were taken from the data set published in reference 11 (GenBank accession number GSE36448), which describes gene regulation by the photoreceptors BLR1, BLR2, and ENV1 in light and darkness upon growth on cellulose. The green background highlights clustering of the wild-type strain and of photoreceptor mutants upon growth on cellulose in panel A, while the yellow background highlights Δ*env1* in LL clusters with soluble carbon sources in panel B.

Cellulose cluster 1 (scaffold 1, 437000 to 526000) contains 2 polyketide synthase genes and *cel61a*. Genes in this cluster are mainly downregulated in light, and the cluster overlaps a CAZyme cluster represented in Fig. 2A of reference 46. Interestingly, this cluster also overlaps cluster “ce” of genes positively correlated with the specific protein production rate (SPPR) as analyzed under conditions of different specific growth rates on lactose (45). Otherwise, except for a few individual genes, no correlation of clustered genes associated with SSPR with genes involved in light responses or in light-dependent regulation of cellulase gene expression was detected.

Cluster 8 (scaffold 6, 101000 to 171000) overlaps a described CAZyme cluster containing *bxl1* (see reference 46 and Fig. 2c therein). Further, we found CAZymes in cluster 6 (scaffold 5, 13000 to 89000; *abf2*) and cluster 12 (scaffold 11, 588000 to 677000; TR_78713 glycoside hydrolase family 72). Besides *abf2*, cluster 6 comprises genes encoding a putative *S*-adenosylmethionine (SAM)-dependent methyl transferase (TR_105242), one predicted zinc-dependent hydrolase potentially involved in detoxification (TR_120877), one lysyl-tRNA synthase (TR_72615), and one uncharacterized gene (TR_105222). In order to learn whether this light-dependent regulation is dependent on the function of photoreceptors, we checked regulation of these genes in the transcriptome data set of photoreceptor mutants upon growth on cellulose (11; GenBank accession number GSE36448). We found that these genes were consistently positively regulated by the BLR1, BLR2, and ENV1 photoreceptors in light, except for *abf2*, which is positively regulated by BLR1 and ENV1 in light but negatively regulated by BLR2 (11).

However, the most interesting light-regulated cluster was regulated positively by light and is located on scaffold 3 (12000 to 94000). It overlaps a described CAZyme cluster (see reference 46 and Supplementary Table S7 therein) and comprises genes encoding one of the two major cellulases (*cel6a* [*cbh2*[ and *cel5a* [*egl2*]), one predicted sugar transporter (TR_56684), one putative urea transporter (TR_56911), and one flavin mononucleotide (FMN)-dependent oxidoreductase (TR_104081). The genes in this cluster are all regulated positively by the BLR1, BLR2, and ENV1 photoreceptors in light (see Fig. S1 in Text S1 in the supplemental material) but not in darkness (11).

10.1128/mSphere.00089-17.1 Text S1  Supplemental text and associated figures. Download Text S1, PDF file, 1.3 MB. Copyright © 2017 Stappler et al. 2017 Stappler et al. This content is distributed under the terms of the Creative Commons Attribution 4.0 International license.

### Genes specific for cellulase-inducing conditions in darkness.

Darkness represents the most relevant condition for biotechnology due to cultivation of fungi in light-tight steel fermenters. Of the 947 genes whose induction is specifically regulated at least 2-fold in darkness (*P* value of 0.01 for false-discovery rate [FDR]), 625 genes showed higher transcript abundance and 322 lower transcript levels under inducing conditions. Investigating the genomic location of these genes, we found that the acetylxylan esterase *axe1* gene (TR_73632), the CBM domain containing auxiliary *cip1* (TR_73638), and the endoglucanase 4 gene (*egl4*) (GH61; TR_73643) form a coregulated cluster that showed more than 5-fold regulation on scaffold 1 (520000 to 526000) (Data Set S4). This cluster overlaps the CAZyme cluster (cluster 1) described in the genome analysis study for *T. reesei* (see reference 46 and Supplementary Table S7 therein). Within the boundaries of this cluster, our data additionally show an upregulated AGC-type kinase (TR_53776) and a gene putatively involved in phosphate metabolism (histidine acid phosphatase/phytase TR_73604).

Another group of neighboring coregulated genes (showing up to 11-fold upregulation) includes *cel1b* (TR_22197) and is located on scaffold 8 (406000 to 425000). It was not identified as a cluster in our previous screening. In addition to *cel1b*, it contains one cytochrome P450 gene (TR_77512), the transcription factor gene *ace3*, and two as-yet-uncharacterized genes (TR_106877 and TR_106879). Additionally, two smaller groups of only 3 genes were found, comprising the cellulase transcription factor *ace2* gene, a major facilitator superfamily (MFS)-type transporter gene (TR_62488), and a predicted protein kinase gene (TR_62181) (scaffold 10, 880000 to 890000, within cluster 17 of the induction-specific genes). The second gene group (scaffold 13, 25000 to 34000) comprises an MFS-encoding gene associated with cellulase signaling (TR_79202), the GPCR TR_63981 gene, and an uncharacterized gene (TR_108663).

Concerning transcription factors, we found that numerous as-yet-uncharacterized genes are coregulated with known cellulase transcription factor genes *ace1*, *ace2*, *ace3*, *xyr1*, and *cre1*, which may hence have related functions. A detailed analysis of this coregulation is provided as supplemental material (Text S1; Data Set S5).

10.1128/mSphere.00089-17.6 DATA SET S5  Coregulation analysis of known cellulase regulators using gene regulation patterns on cellulose, lactose, sophorose, glucose, and glycerol in light and darkness. Download DATA SET S5, XLSX file, 0.1 MB. Copyright © 2017 Stappler et al. 2017 Stappler et al. This content is distributed under the terms of the Creative Commons Attribution 4.0 International license.

Genes significantly regulated only in darkness (induction specific, dark specific) and not in light include 16 CAZyme-encoding genes, among them *egl3* (*cel12a*) and the predicted oxidoreductase TR_56840 gene (both with more than 8-fold positive regulation) as well as *bgl1* (*cel3a*), *egl5* (*cel45a*), *cel5b*, and *bgn1*. Additionally, the 2 hydrophobin genes *hfb2* and *hfb5*, 4 genes involved in oxidative/radical mechanisms, 8 protease genes, 11 transporter genes, and 4 transcription factor genes, including the cellulase transcription factor gene *ace2* and 4 genes predicted to have functions in oxidative and radical mechanisms, were found in this gene group. In addition, interestingly, the *lae1* methyltransferase gene, which is essential for cellulase gene expression (48), shows statistically significant induction-specific regulation only in darkness.

In order to identify the genes most crucial for cellulase-inducing conditions in darkness, which reflect conditions encountered by *T. reesei* in industrial fermenters, we increased the stringency of our statistical analysis to reflect *P* values of 0.001 and at least 5-fold regulation (Data Set S2). A total of 150 genes remained in this data set. Downregulation was observed for 23 genes, and 127 were upregulated. The latter included 21 glycoside hydrolase genes corresponding to all expected highly regulated cellulases, *axe1*, genes encoding two CBM family proteins (one of them being *cip1*), genes encoding six transporters, and three genes involved in oxidative processes; however, two protease genes (TR_22459 and TR_67761) were also found in this strongly regulated gene set. Among the downregulated genes, we found two encoding LysP amino acid permeases (TR_67806 and TR_109122), two encoding PTH11-type GPCRs (TR_122824 and TR_39587) with more than 25-fold downregulation, and a gene encoding a family 90 glycosyl transferase.

### Genes specific for cellulase-inducing conditions in light.

Of the 907 genes showing more than 2-fold regulation (*P* value, <0.01 [FDR]) in light, 684 were upregulated under inducing conditions (induction specific, light specific) and 223 were downregulated under those conditions (Fig. 1B; Data Set S2). Among the genes downregulated in light, no groups of coregulated and closely neighboring genes were found. The cluster comprising *axe1* described above is upregulated in light. Additionally, we found among the genes upregulated in light a group of induction-specific genes within cluster 13 around the gene encoding the sugar transporter TR_3405, which was characterized as a lactose permease essential for cellulase induction on lactose (34). In the close vicinity of TR_3405, we found genes encoding a dehydrogenase (TR_106164; induction-specific regulation) and an oligopeptide transporter (TR_59364 induction and light-specific regulation).

Genes showing induction-specific regulation only in light and not in darkness resemble those showing induction-specific regulation in darkness but appear to be complementary: they include genes encoding 9 proteases, 10 genes encoding ribosomal proteins, 6 genes encoding transcription factors and a CreC homologue (TR_64608), and a gene encoding a WD40 repeat protein, which is assumed to contribute to regulation of the CRE1 carbon catabolite regulator. Additionally, 10 genes encoding ribosomal proteins and 10 transporters, including the glucose transporter *hxt1*, are induction specific in light, as are 5 genes involved in Ca-dependent signaling and the gene encoding the regulatory subunit of protein kinase A (PKAr1). Moreover, 14 CAZyme-encoding genes, including a gene encoding a candidate trehalase (TR_123226), *bgl3i*, *cel3e*, *cel5d*, and the chitinase genes *chi18-18* and *chi18-9* as well as two genes associated with secretion (TR_81263 and TR_123015), are regulated only in light in an induction-specific manner. Together with the genes specifically regulated under inducing conditions in darkness, these genes reflect intricate regulation of a portion of degradative enzymes and the uptake/utilization of the available substrate under conditions of daylight and darkness.

### Genes specific for cellulase-inducing conditions independent of light.

Differential regulation under inducing conditions was found in light and darkness (light-independent induction specificity) for 530 genes. Of them, 383 were upregulated and 147 were downregulated (Data Set S2; Fig. 1B).

A total of 47 CAZyme-encoding genes were regulated under inducing conditions in light and darkness, including those encoding the major cellulases. Strong (84-fold) upregulation was found for hydrophobin gene *hfb3* in light and darkness under inducing conditions. Four genes involved in oxidative and radical mechanisms were in this gene group, with three of them, including the gene encoding catalase C, being upregulated around 10-fold. Moreover, upregulation of seven proteases was observed in light and darkness. Furthermore, we found 15 signaling genes, 14 transporter-encoding genes, and 4 genes involved in sulfur metabolism as well as 9 transcription factor genes, including *xyr1*, among the genes regulated under inducing conditions in light and darkness. The two beta glucosidase genes *cel1a* and *cel1b* (TR_120749 and TR_22197), which were recently shown to be essential for cellulase induction on lactose (49), are strongly (more than 11-fold) upregulated under inducing conditions in light and darkness. In light, interestingly, both are considerably regulated by ENV1 (11), indicating that ENV1 has an important function for efficient induction of plant cell wall degradation in light.

### Functions regulated under inducing conditions in light and darkness.

In summary, our functional category analysis showed that the crucial function of C-compound and carbohydrate metabolism, including sugar, polyol, and carboxylate metabolism as well as polysaccharide metabolism, is significantly enriched among genes with induction-specific regulation in light and darkness (Data Set S6). General metabolic functions, including amino acid metabolism, energy production, protein synthesis, and cellular transport, are enriched among downregulated genes. However, we do see distinct functions that are enriched either in light or in darkness and functions enriched in light and darkness to different extents. In some cases, clear functional differences were observed between light and darkness. For example, secondary metabolism was enriched among functions of downregulated genes in darkness but not in light. In contrast, we found genes assigned to the same functional category among the genes regulated under inducing conditions only in darkness and only in light, which suggests that different genes are applied for the same function in light and darkness. For a detailed analysis of the functional categories, see Text S1 and Data Set S6. Analyzing the shift in total transcripts produced in light compared to darkness, i.e., the resources invested in light or darkness on cellulose, we found a strong enrichment in metabolism in general, in particular, sulfur metabolism and C-compound and carbohydrate metabolism as well as polysaccharide metabolism. This result suggests the high relevance of the increased abundance of enzyme-encoding transcripts in light. A detailed analysis is provided in the supplemental material (Text S1; Data Set S7). Genes associated with repression of cellulase gene expression upon growth on glucose versus growth on glycerol along with their functional categories are outlined in the supplemental material (Text S1; Data Set S8).

10.1128/mSphere.00089-17.7 DATA SET S6  Functional category analysis of induction-specific genes in light and darkness. Download DATA SET S6, XLSX file, 0.2 MB. Copyright © 2017 Stappler et al. 2017 Stappler et al. This content is distributed under the terms of the Creative Commons Attribution 4.0 International license.

10.1128/mSphere.00089-17.8 DATA SET S7  Analysis of genes reflecting devotion of resources on cellulose, sophorose, and glucose. Download DATA SET S7, XLSX file, 0.04 MB. Copyright © 2017 Stappler et al. 2017 Stappler et al. This content is distributed under the terms of the Creative Commons Attribution 4.0 International license.

10.1128/mSphere.00089-17.9 DATA SET S8  Genes differentially regulated between growth on glucose and growth on glycerol reflecting repression-specific regulation. Download DATA SET S8, XLSX file, 0.04 MB. Copyright © 2017 Stappler et al. 2017 Stappler et al. This content is distributed under the terms of the Creative Commons Attribution 4.0 International license.

### Induced genes not regulated by photoreceptors or in a manner dependent on light.

The BLR1, BLR2, and ENV1 photoreceptors as well as their homologues in *N. crassa* were shown to be involved in regulation of expression of cellulolytic enzymes (9, 12, 50). We were therefore interested in determining the extent to which they regulate cellulase induction-specific genes (induction specific, light independent). Of the 1,324 genes regulated specifically under inducing conditions in light or in darkness or both, 753 genes were regulated by BLR1, BLR2, or ENV1 (Fig. 1C; Data Set S2). Of the 571 remaining genes, 353 still showed different induction-specific gene regulation patterns in light versus darkness, hence indicating alternative light-dependent regulation. Only 218 genes (16.5%) were found to be induction specific in light and darkness and not regulated by the photoreceptors. Of the 145 genes upregulated in this gene set, only those corresponding to functions in vacuolar protein degradation mechanisms (*P* value 1.56e−03) and in transmembrane- and cAMP-dependent signal transduction mechanisms (*P* values of around 3e−03) were significantly enriched. This gene set comprised three glycoside hydrolase genes of families 65, 76, and 125, the phosphodiesterase 1 gene (*pde1*; TR_3873), seven kinase genes, and two GTPase regulator genes. Interestingly, a *dap1* orthologue encoding a protein involved in DNA methylation is also among these genes.

Enrichment in functions of metabolism (*P* value 4.28e−03), especially amino acid metabolism (*P* value 8.65e−04), transfer of activated C1 groups (*P* value 3.79e−03), and rRNA synthesis and processing (*P* values <2e−05) as well as protein synthesis (*P* value 1.31e−04) and ribosome biogenesis (*P* value 3.35e−05), was detected in the gene set downregulated under inducing conditions independently of light or photoreceptors (73 genes). Additionally, we found enrichment in transport functions (*P* value 4.28e−04), including C-compound and carbohydrate transport (*P* value 1.06e−03), amino acid transport (*P* value 1.33e−05), and heat shock response (*P* value 3.37e−04). Among these genes, none encoding glycoside hydrolase were found, but one encoding a glycosyltransferase potentially involved in glycosylation (TR_4561) was found. In summary, neither the known cellulase regulators nor the glycoside hydrolases or proteins involved in oxidative degradation of cellulose were found to be regulated independently of light or photoreceptors under inducing conditions.

### Contribution of photoreceptors to induction-specific upregulation of genes.

Of the three photoreceptors of *T. reesei*, BLR1 and BLR2 represent GATA type transcription factors (9), which are likely to contribute to induction-specific gene regulation based on the enrichment of metabolism-related genes among their targets (11). In order to evaluate the contribution of the photoreceptors to upregulation of induction-specific genes, we compared the genes upregulated in light and darkness, those upregulated only in light, and those upregulated only in darkness with the targets of photoreceptors (11). Of the 383 genes upregulated under inducing conditions in light and darkness, 61 genes are positively regulated by BLR1 in light (11) (Data Set S8). Among these genes, those associated with functions in metabolism (*P* value 2.63e−04), and in C-compound and carbohydrate metabolism (*P* value 7.54e−08) in particular, are significantly enriched, with strongest enrichment in polysaccharide metabolism (*P* value 1.46e−11). Twenty-one CAZyme-encoding genes, including *cbh1* (*cel7a*), *cbh2* (*cel6a*), *egl1* (*cel7b*), *egl2* (*cel5a*), *cel61b*, *xyn2*, and *bxl1*, are positively regulated in light by BLR1, along with the *xyr1* cellulase regulator gene. Of these 61 genes, only 5 largely uncharacterized genes are positively regulated by BLR1 in darkness and none of the genes regulated under inducing conditions in light and darkness are positively regulated by BLR1 only in darkness. Thirty-one of the genes upregulated under inducing conditions in light and darkness were positively regulated by BLR2 in light, and 24 overlapped those positively regulated by BLR1 in light (Data Set S2). For regulation of these 24 genes, BLR1 and BLR2 can be expected to act as a complex. Among these 24 genes are 5 CAZy-encoding genes, including *xyn2* and *abf3*. Twenty-two of the genes upregulated under inducing conditions only in darkness are positively regulated by BLR1 in light; among them, *egl3* (*cel12a*), *bgl1* (*cel3a*), and *egl5* (*cel45a*) and 10 largely uncharacterized genes are regulated positively by BLR1 in darkness (Data Set S2).

Seventy-seven genes among those regulated positively under inducing conditions only in light were also regulated positively by BLR1 in light (Data Set S2). These genes included 5 CAZy-encoding genes, among them *cel3e*, a trehalase-encoding gene, and 2 putative phospholipase C-encoding genes (*plc-e* and TR_61746). This analysis showed that with respect to regulation of substrate degradation, photoreceptors clearly contribute to regulation of induction-specific genes, including enzyme-encoding genes, in light and darkness (Data Set S2). Therefore, BLR1 has a broader influence on induction-specific gene regulation than BLR2 and exerts its function predominantly in light.

### Genome-wide regulation patterns associated with nutrient and light signaling.

Our results revealed that most of the induction-specific genes show different regulation patterns in light and darkness or are subject to regulation by photoreceptors or both. Moreover, we observed light-dependent enrichment in several functions in different gene sets. Hence, we wanted to test if this functional specialization is also present in genome-wide regulation patterns on different carbon sources versus regulation by photoreceptors upon growth on cellulose. Or, in other words, we asked whether regulation by the photoreceptors BLR1, BLR2, and ENV1 can interfere with (i.e., break) carbon source-specific regulation and light-specific regulation. Therefore, we reanalyzed the transcriptome data from our present study together with those from photoreceptor mutants grown on cellulose in light and darkness (11; GenBank accession number GSE36448). Hierarchical cluster analysis clearly showed clustering on inducing carbon sources versus glucose in light and darkness (Fig. 2A). Induction of cellulase gene expression by sophorose is still possible in the presence of glycerol, which is in accordance with the glycerol patterns being associated with glucose (darkness) as well as with lactose and sophorose (light). Cellulose is clearly separated from all soluble carbon sources. The genome-wide regulation patterns in photoreceptor mutants in light are divergent from those present in darkness; in particular, the strain lacking ENV1 in light is separated from the other strains growing on cellulose. However, the induction specificity of regulation patterns is not broken (Fig. 2A).

Using specific gene groups for this clustering revealed that for glycoside hydrolases, the Δ*env1* mutant clustered with the strains corresponding to soluble inducing carbon sources in light and not with strains grown on cellulose anymore. For this gene group, photoreceptor mutants clearly clustered with the wild type in darkness but not with the wild type in light (Fig. 2B). Essentially similar results were found for genes encoding proteases, transcription factors, and heterotrimeric G-protein signaling and for genes involved in glycosylation, secretion, signaling, and transport but not for mitochondrial protein-encoding or ribosomal protein-encoding genes. Consequently, hierarchical clustering shows that transcript patterns of mutants with mutations in *blr1* and *blr2* still show light-dependent differences. This supports the idea of a function of BLR1 and BLR2 in light-dependent gene regulation on cellulose. Additionally, the fact that light-grown cultures of these mutants still clustered with light-grown cultures of the wild type and not with the dark-grown cultures indicates that a light signal is still transmitted despite the absence of these photoreceptors. Nevertheless, photoreceptors do not break carbon source-dependent signaling. In the absence of ENV1, cellulose-specific sensing is lost in light, albeit the distinction between inducing and noninducing carbon sources remains even in such a mutant.

### Indications for the relevance of surface sensing for gene regulation.

Substrate sensing has been suggested also to involve surface sensing of degradable plant material and is hence important for regulation of plant cell wall-degrading enzymes. A PTH11-type GPCR emerged as a candidate for such a function in *Aspergillus niger* (51). Therefore, we analyzed which genes are enhanced upon growth on the insoluble inducing carbon source cellulose versus the two soluble inducing carbon sources sophorose and lactose. Indeed, our data indicate that the types of gene regulation differ between growth on soluble carbon sources and growth on insoluble carbon sources (Data Set S9). Under conditions of growth in the presence of soluble inducing carbon sources, we found a strong downregulation of swollenin, which destabilizes cellulosic material without liberation of reducing sugars (52). Further, *cip1* and *cip2*, auxiliary cellulose binding genes important for efficient utilization of cellulose (28, 53), were downregulated under these conditions in light. Also, the class II hydrophobin gene *hfb3* was downregulated in light. Hydrophobins are assumed to support attachment of hydrolytic enzymes to their substrates (54). The *T. atroviride* homologue of *hfb3*, *hfb-5a* (55), was previously found to be regulated by light and by the photoreceptors BLR1 and BLR2 (56). Moreover, 8 predicted G-protein-coupled receptors, including four PTH11-like GPCRs (TR_121990, TR_124113, TR_27992, and TR_76763) and two RGS domain-containing GPCRs related to *Aspergillus* GrkA and GrkB (class VI; TR_37525 and TR_63981) were downregulated on soluble inducing carbon sources in light. In darkness, only 16 glycoside hydrolases are negatively regulated under these conditions, as well as 7 genes involved in secretion and 4 GPCR genes (TR_69500/PTH11-like, TR_55561/PTH11-like, TR_120238/class XIII, and TR_27948/class XIII), with TR_27948 being downregulated in light and darkness, albeit only 2-fold to 3-fold. Also, 35 glycoside hydrolases showed lower transcript levels on lactose and sophorose than on cellulose in light, and, accordingly, 8 genes involved in secretion also followed this trend. Interestingly, of the 114 genes downregulated in the Δ*env1* and Δ*acy1* mutants which are assumed to represent cAMP-dependent targets of ENV1 in light (18), 88 (77%) were also downregulated on soluble inducing carbon sources versus cellulose in light (Data Set S9). Hence, the cAMP-related output of regulation by ENV1 could be aimed at surface-sensing functions, which would be in accordance with the loss of clustering seen with cellulose-grown cultures in the Δ*env1* mutant (Fig. 2). The striking difference in potential surface-sensing GPCRs between light and darkness suggests substrate recognition on a surface, which is likely in light, whereas after penetration of the substrate (darkness), surface sensing has lower priority. Alternatively, these GPCRs may also respond to the presence of a cellulose-derived compound other than sophorose that signals the necessity to enzymatically degrade cellulose.

10.1128/mSphere.00089-17.10 DATA SET S9  Genes associated with surface sensing under inducing conditions and the relevance of cAMP signaling as influenced by ENV1. Download DATA SET S9, XLS file, 1.1 MB. Copyright © 2017 Stappler et al. 2017 Stappler et al. This content is distributed under the terms of the Creative Commons Attribution 4.0 International license.

### G-protein-coupled receptors putatively involved in cellulose sensing.

**FIG 3:**
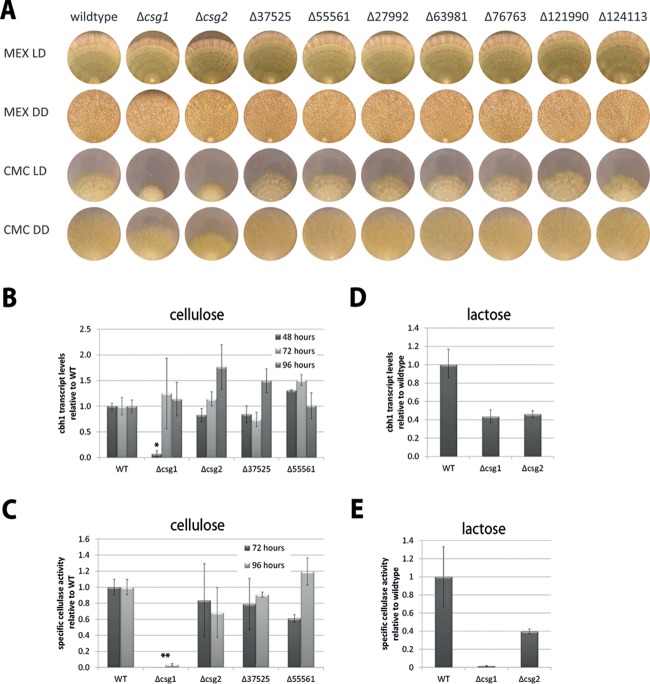
Analysis of GPCR deletion strains. (A) Growth of GPCR deletion mutants on rich medium versus minimal medium with cellulose as the carbon source. Strains were grown on 3% (wt/vol) malt extract agar plates (MEX) or Mandels-Andreotti agar plates with 1% (wt/vol) carboxymethylcellulose (CMC) for 7 days at room temperature in daylight (LD) or constant darkness (DD). Inoculation was done at the edge of the plates to allow several days of measurement. A picture representative of results from 3 biological replicates is shown. (B) *cbh1* transcript levels of the Δ*csg1*, Δ*csg2*, Δ37525, and Δ55561 mutants were determined by quantitative RT-PCR after growth on cellulose in constant darkness after 48, 72, and 96 h and are shown relative to wild-type (WT) QM6a results (*, *P* ≤ 0.01). (C) Specific cellulase activity in culture filtrates after growth on cellulose for 72 or 96 h in constant darkness. Activity data are given relative to that of the wild-type QM6a strain (*, *P* ≤ 0.01; **, *P* ≤ 0.001). (D) *cbh1* transcript levels of the Δ*csg1* and Δ*csg2* mutants after growth on lactose in constant darkness after 40 h are shown relative to the wild-type results. (E) Specific cellulase activity in culture filtrates after growth on lactose for 40 h in constant darkness. Activity is given relative to the wild-type results.

We selected 9 of the regulated genes for deletion and phenotypic analysis (Fig. 3A). Hyphal extension rates were analyzed on complex media and minimal media with carboxymethylcellulose (CMC) as the carbon source (Fig. S4 in Text S1). Deletion of TR_27948 and TR_120238 (both class XIII) caused clearly decreased growth specifically on CMC (decrease of the hyphal extension rate in TR_27948 to 82% ± 1.1% [*P* value = ≤0.001] and in TR_120238 to 83% ± 2.0% [*P* value = ≤0.002] compared to the wild type) but not on malt extract media (Fig. 3A; Fig. S4 in Text S1). Interestingly, these two GPCRs belong to the DUF300 domain-containing class XIII GPCRs (1), for which no function had been defined so far. Hence, they were selected for further analysis and named *csg1* and *csg2* (cellulose-specific GPCR 1 and 2). CSG1 and CSG2 represent all members of class XIII GPCRs in *T. reesei*. Additionally, we chose strains TR_37525 and TR_55561 because they grew better on carboxymethylcellulose than the wild-type strain (growth of TR_37525 increased to 126% ± 3.0% [*P* value = <0.001], and growth of TR_55561 increased to 119% ± 1.1% [*P* value = <0.001]). The deletion strains representing the four genes selected for further analysis were confirmed to have only one integrated copy of the deletion cassette.

Upon growth on microcrystalline cellulose in shake flask cultures, deletion of *csg1* considerably decreased growth to less than 50% of the wild-type level, while the Δ*csg2* mutant grew normally. The Δ37525 and Δ55561 mutants grew even faster than the wild-type strain at 72 h (more than 1.5-fold), but growth decreased to wild-type levels thereafter (96 h) (data not shown). Analysis of *cbh1* transcript levels in these deletion strains revealed delayed induction for the Δ*csg1*, Δ*csg2*, and Δ37525 mutants compared to the wild-type results; however, the transcript levels increased to wild-type levels and above at later time points (Fig. 3B). Nevertheless, we found that these *cbh1* levels did not translate to similar specific cellulase activities (Fig. 3C), which decreased strongly for the Δ*csg1* mutant, decreased moderately for the Δ*csg2* and Δ37525 mutants, and even increased for the Δ55561 mutant. In order to confirm the defect of the Δ*csg1* mutant in specific cellulase activity, we crossed the Δ*csg1* strain with female fertile MAT1-1 strain FF1 (57) and found that, in progeny lacking *csg1*, the specific cellulase activity in the progeny again dropped to 5.1% ± 1% of that seen with parental strain FF1. We then cultivated the Δ*csg1* and Δ*csg2* mutants on lactose, a carbon source which induces cellulase gene expression, although cellulases are not needed for growth on lactose. This analysis enabled evaluation of the effect of *csg1* and *csg2* deletion on cellulase gene expression independently of a potential growth defect caused by lack of degrading enzymes. *cbh1* transcript levels on lactose were decreased to roughly 40% of wild-type levels (Fig. 3D). While the decrease in specific cellulase activity corresponded to the decrease in *cbh1* transcript levels for the Δ*csg2* mutant, the specific cellulase activity in the Δ*csg1* mutant dropped to less than 2% of the wild-type activity (Fig. 3E).

With the same rationale, we cultivated the strains on cellulose with the addition of glycerol to enable growth despite potentially abolished cellulase formation. Previously, induction of cellulases by the natural inducer sophorose was shown not to be perturbed in the presence of glycerol (30, 58). We found that both the wild-type strain and the *csg* mutants form abundant biomass on medium containing cellulose and glycerol but that no cellulases were detectable, which is in agreement with the previous assumption that glycerol represents a repressing carbon source, albeit our findings show that this applies only in combination with cellulose.

We conclude that CSG1 is essential for cellulase regulation and that its cognate signaling pathway likely interferes with high-level production of cellulolytic enzymes at a posttranscriptional level. CSG2 appears to be relevant but of minor importance for regulation of cellulase gene expression. The corresponding results on cellulose and lactose indicate that CSG1 and CSG2 sense chemical compounds (glucose) and that the observed surface sensing is achieved by different components.

## DISCUSSION

Like most higher eukaryotes, including humans, fungi react to the different levels of light present during the day and at night and adjust their metabolism both following light pulses (35) and according to their circadian rhythm (59). Anabolic and catabolic functions are precisely regulated at dusk and dawn (59, 60), and even short light pulses result in considerable alterations in gene regulation (40). Measures that are protective against UV light, desiccation on the (illuminated) surface, or altered oxidative stress levels (36) explain in part the adjustment of gene regulation during the day and at night. However, why enzyme expression for substrate degradation changes in light and darkness, as shown previously (8, 10, 11), although the substrate itself remains the same, remains to be elucidated.

In order to gain more insight into light-dependent and substrate-specific gene regulation, we chose 5 carbon sources with different characteristics (inducing, noninducing, repressing, and soluble or insoluble) for our study and investigated gene regulation in light and darkness. We confirmed considerable metabolic adaptations in light and darkness and also gene regulation specific for cellulase-inducing conditions showing specific alterations. Analyzing induction-specific regulation in light and darkness, we found that rather than representing whole functional groups, the genes within a group were differentially regulated. This finding correlates with the fact that in several functional groups, including those composed of glycoside hydrolase-encoding genes and, in particular, ribosomal protein-encoding genes, we detected subgroups with higher relevance in light and others more suited for growth in darkness. Also, important cellulase transcription factors are regulated in a light-dependent manner. In many cases, photoreceptors influence this gene regulation and only relatively few genes are induction specific without a relevance of light or photoreceptors.

Interestingly, genes corresponding to light-dependent regulation on cellulose are not randomly distributed in the genome and many CAZyme clusters overlap light-regulated clusters on cellulose or clusters corresponding to regulation of induction-specific genes or both. This finding is in accordance with studies showing epigenetic regulation of cellulase gene expression (58, 61). Additionally, it also strengthens the idea of a connection between nutrient-dependent regulation of gene expression and light-dependent regulation. Further support for this connection comes from data revealing a considerable increase in transcript abundance of genes involved in carbon metabolism and transport, which are among those most strongly upregulated in light.

With respect to carbon-specific regulation, we found that sensing of inducing carbon sources is strongly reflected in gene regulation and is also adjusted in light and darkness. An involvement of the heterotrimeric G-protein pathway in cellulase regulation that takes place in a light-dependent manner has been shown for the G-alpha subunits GNA1 and GNA3 (15, 17). Also, genome-wide analysis of the beta and gamma subunits revealed considerable regulation of CAZyme-encoding genes (10). Our study results now indicate that predicted G-protein-coupled receptors are also involved in regulation of cellulase gene expression. This is in accordance with findings in *N. crassa*, where the PTH11 class GPCRs GPR-32, GPR-36, and GPR39 have a negative effect on growth in the presence of cellulose. However, no GPCR with a positive effect on growth on cellulose was found for the class XIII GPCRs of *N. crassa* (22).

**FIG 4:**
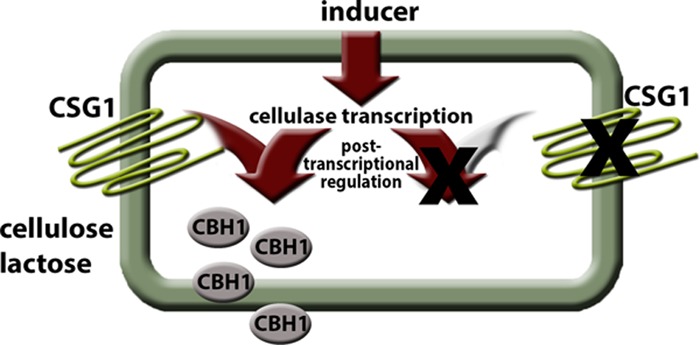
Model of CSG1 function. Activation of cellulase gene transcription occurs in response to the presence of an inducer. CSG1 senses the presence of cellulose or lactose, likely represented by their common building block glucose, indicating the need for enzymatic degradation to enable uptake of nutrients, and hence stimulates posttranscriptional processes and/or translation and secretion of cellulolytic enzymes. In the absence of CSG1, the signal initiating production or activation of cellulases is not transmitted and production or function of plant cell wall-degrading enzymes remains at very low basal levels.

Our results show a positive effect on growth on cellulose for CSG1. Interestingly, its major impact was not on transcription but rather on a posttranscriptional step of cellulase regulation on cellulose, including altered protein turnover or even activity (Fig. 4). A similar effect occurs on lactose, which, due to the formation of an extracellular beta-galactosidase (62), is (at least in part) cleaved to glucose and galactose prior to entering the cell. Hence, the building block glucose as derived from cellulose and lactose is likely to be the ligand and signal of CSG1, which initiates the posttranscriptional steps required for cellulase enzyme production. We conclude that the pathway regulating cellulase formation comprises two sections: one receives the inducing signal, likely in response to uptake of the inducer, and the second initiates cellulase formation in a manner dependent on the signal transmitted by CSG1. This bipartite regulation enables efficient use of resources needed for substrate degradation (upon initiation of protein formation), despite providing for a mechanism allowing elevated cellulase transcript levels facilitating rapid response to the sudden need of protein formation. The existence of such a mechanism is likely due to a beneficial economical effect necessary for the cell to avoid unnecessary protein production. Regulatory processes ensuring that a transcript is synthetized but that the protein is not or that it is synthetized at a lower level are assumed to be required to enable a fast reaction to changes in the environment (63).

Data from previous studies investigating the relationship of the abundance and regulation of the transcript to the abundance of the respective proteins support the hypothesis that additional levels of regulation impact gene regulation in *T. reesei* and that such is also the case under cellulase-inducing conditions (45). A limitation in the protein secretion capacity and in the onset of the unfolded protein response was proposed to contribute to such differences (64). Our findings are in agreement with the widespread posttranscriptional regulation of metabolic potential as reported recently (60) and now indicate that there are mechanisms that can interfere with cellulase production at the posttranscriptional level.

For *N. crassa*, considerable posttranscriptional regulation occurs in response to cellulosic material, which includes important cellulolytic enzymes (65), and was also confirmed for circadian gene regulation, which is impacted by light (60).

Investigation of constitutive activation of GNA1 and GNA3 revealed strong light-dependent regulation of cellulase expression by both factors, but the presence of an inducer was still required for the formation of cellulases (15, 17). Consequently, the signal for induction of cellulase gene transcription must be transmitted via routes independent of the heterotrimeric G-protein pathway, and the data mainly support the idea of the adjustment of transcript levels as a prerequisite for high-level enzyme production. If these enzymes are not needed (because the soluble carbon source can be utilized without their degradative function), their biosynthesis would represent a waste of cellular resources, which would justify a function like that of CSG1—to enable enzyme production upon sensing of cellulose by initiation/enhancement of translation. Nevertheless, as cellulases are still produced upon growth on lactose and sophorose, albeit at lower levels, either this mechanism is leaky or the coordination with transcription might be specific to cellulose degradation.

Our two-dimensional analysis confirmed that light is an important regulator of enzyme gene expression not only on cellulose but more generally under inducing conditions in *T. reesei*. We also found that light has lower priority than the presence of inducing carbon sources. Additionally, the finding of a G-protein-coupled receptor involved in posttranscriptional regulation now indicates that the previous hypothesis of cellulase regulation at the level of transcription has to be reconsidered. Rather, our data reveal that *T. reesei* applies signaling mechanisms to decide on the necessity (surface sensing or sensing of a cellulose-derived product other than sophorose), nature (with regard to which enzymes are suitable in the presence of light versus darkness), and quantity of enzymes under given environmental circumstances. It can further be concluded that controlled light conditions are advisable for screenings to achieve reproducibly high performance of selected production strains during upscaling. Further investigation in this direction will significantly contribute to knowledge-based industrial strain improvement by considering light effects during screening and by exploiting the connected gene regulation for improved production in the dark environment of an industrial fermenter.

## MATERIALS AND METHODS

### Strains and culture conditions.

In this study, strain QM9414 and strain QM6a and its derivative strains QM6aΔ*ku80* and QM6aΔ*mus53* (66) were used. For crossing with the Δ*csg1* strain, we used female fertile strain FF1, a derivative of QM6a obtained by backcrossing (57). These strains were used as controls in every experiment along with the mentioned mutants of similar background. For propagation and hyphal extension analysis, strains were grown on 3% (wt/vol) malt extract-agar plates (Merck, Darmstadt, Germany). For quantitative reverse transcription-PCR (qRT-PCR) analysis, biomass determinations, and determinations of cellulase activity, strains were grown in liquid culture in 100 ml Mandels-Andreotti (67) minimal medium supplemented with 0.1% (wt/vol) peptone (Roth, Karlsruhe, Germany) to induce germination and with 1% (wt/vol) microcrystalline cellulose (Alfa Aesar, Karlsruhe, Germany) with or without addition of 1% (wt/vol) glycerol (Roth), or with 1% (wt/vol) lactose (Roth) as a carbon source. Strains were harvested after 48, 72, or 96 h of growth on microcrystalline cellulose, after 72 h of growth on cellulose in combination with glycerol, and after 40 h of growth on lactose at 28°C on a rotary shaker (200 rpm). For transcriptional profiling, we assessed mycelia grown on different carbon sources at time points of active growth and cellulase gene expression as revealed in earlier studies (8, 16, 39). Specifically, we used 72 h for cellulose, 24 h for glycerol, 20 h for glucose, and 30 h for lactose and the replacement of sophorose was done for 5 h after a 24-h preculture on glycerol. Strains were grown either in constant illumination (Osram L 18W/835 fluorescent bulbs) (1,800 lx) or in constant darkness. Plate cultures for inoculum preparation were performed for 10 days in constant darkness in order to avoid interference of light effects or circadian rhythms.

### Phenotypic analysis.

Malt extract-agar plates (3% [wt/vol]) and Mandels-Andreotti agar plates with 1% (wt/vol) carboxymethylcellulose (Sigma, USA) were inoculated with spore solution and incubated at room temperature in daylight or constant darkness. Pictures were taken after 7 days. Strains were grown in three biological replicates. For analysis of hyphal extension, 3% (wt/vol) malt extract-agar plates and Mandels-Andreotti agar plates with 1% (wt/vol) carboxymethylcellulose (Sigma, USA) were inoculated with spore solution and incubated at room temperature in daylight. Hyphal extension was measured every 24 h. Strains were grown in three biological replicates.

### Construction of *T. reesei* deletion strains and copy number determination.

Deletion vectors were constructed by yeast recombination cloning using primers for flanking sequences for creating *hph* marker constructs (see Supplementary File S4 of reference 68) as described earlier (68). Protoplast transformation was used for deleting genes in the QM6aΔ*ku80* strain or the QM6aΔ*mus53* strain. Absence of the open reading frames of target genes was confirmed by PCR. We used at least two different deletion strains per gene for analysis of cellulase expression, and copy number determinations revealed that they had only one copy of the deletion cassette integrated. Copy number determination was performed as described earlier (10). Briefly, copy number was determined by quantitative PCR (qPCR) using genomic DNA. *l6e* was used as the reference gene/single-copy gene, and the following two strains with known copy numbers were used as controls: the GNA3QLE strain (69), which contains one copy of the *hph* marker cassette, and the Δ*gng1* mutant (10), which contains three copies of the *hph* cassette. We found that the strains used for further analysis in our study all contained only a single integration of the *hph* marker cassette and no ectopic integration.

### RNA isolation, cDNA synthesis, and quantitative real-time PCR.

Strains were grown as described above, and mycelia were harvested by filtration, briefly washed with Mandels-Andreotti media lacking a carbon source, and snap-frozen in liquid nitrogen. For cultures grown in constant darkness, harvesting was done under red safety light. Extraction of total RNA was carried out as described in reference 69 using an RNeasy Plant minikit (Qiagen, Hilden, Germany). RNA quality was checked by agarose gel electrophoresis and by the use of an Agilent 2100 Bioanalyzer with an Agilent RNA 6000 Nano Chip. Samples with an RNA integrity number (RIN) value above 9 were used for further analyses. Total RNA (1 µg) was treated with DNase I (Thermo Fisher, Waltham, MA). Reverse transcription was performed using a RevertAID H minus first-strand cDNA synthesis kit (Thermo Fisher, Waltham, MA) and oligo(dT)_18_ primers. qRT-PCRs were performed as described previously (69) on CFX96 Real-Time cyclers (Bio-Rad). The final PCR mixture contained 10 µl GoTaq qPCR master mix (Promega, Madison, WI), 5 µl cDNA (diluted 1:20), and 200 nM forward and reverse primers in a total volume of 20 µl. Primers for *cbh1* and *L6e* (reference gene) were used as given in reference 69. At least two different strains for every deletion with two biological replicates per strain/transformant (4 replicates total) and three technical replicates were considered. Data analysis was done using qbase+ (Biogazelle) software.

### Transcriptome analysis.

Total RNA was isolated as described above, and quality constraints similar to those used with qRT-PCR were applied. For transcriptome analysis, we used the gene expression full service for custom arrays as provided by Roche-Nimblegen (Madison, WI, USA). Two independent biological replicates were considered for all analyses, and a threshold of 2-fold with a *P* value of 0.01 (FDR corrected) was applied for differential gene regulation (analysis of variance [ANOVA] statistics; see below). The individual samples and replicates were combined for analysis according to the specific scientific issues such that 4 or more (up to 10) replicates were in each combined group for comparative analysis. Coefficients of correlation between biological replicates under a given set of conditions (carbon source/light status) were on average 0.95 (chi square association) with no decrease below 0.93 for any condition.

### Bioinformatic analyses.

Bioinformatic analysis of transcriptome data was done using Partek Genomics suite 6.6 (Partek Inc., St. Louis, MO), which uses ANOVA for evaluation of statistically significant differentially expressed genes across multiple data sets. A false-discovery rate (FDR) control was used to correct for multiple comparisons in order to control the expected proportion of incorrectly rejected false discoveries (null hypothesis). Statistical evaluation of GPCR mutant analysis was performed using the open source software package PSPP for Windows. For hierarchical clustering and coregulation analyses, HCE3.5 open source software (70) was used with default settings but applying the Poisson correlation coefficient as the similarity/distance measure. In order to ensure the statistical significance of hierarchical clustering results, we removed data with low signal strength, which tend to higher variations, and we additionally removed all gene data with an average standard deviation between replicates of above 20%. The open source software REEF (71) was used for genomic cluster analysis (window width, 200,000; shift, 30,000; *Q* value, 0.1; minimum number of genes in cluster, 3). Adjacent overlapping clusters were fused manually. Functional category analysis was done with the MIPS Functional Catalogue tool in the latest version available in May 2014 (http://mips.helmholtz-muenchen.de/funcatDB/) (72).

### Cellulase activity and biomass determination.

Mycelium from cultivation in liquid media (see above) was snap-frozen in liquid nitrogen and ground in a Retsch mill (Retsch MM301; Retsch, Haan, Germany) in precooled jars for 30 s with an oscillation frequency of 30 Hz and transferred to tubes containing 5 ml 0.1 M NaOH. Samples were sonicated three times for 30 s and incubated for 3 h at room temperature. After centrifugation for 10 min at 3,220 × *g*, the protein concentration of the supernatant reflecting biomass content was measured by the Bradford method according to standard methods. Endo-1,4-β-d-glucanase activity in the culture filtrates was analyzed using an azo-CM-cellulose kit (S-ACMC-L; Megazyme, Wicklow, Ireland) according to the manufacturer’s instructions. For analysis of specific cellulase activity, measured enzyme activity was correlated to biomass. At least two different strains for every deletion, with two biological replicates per strain/transformant (4 replicates total) and two technical replicates, were considered.

### Accession number(s).

Data were deposited in the NCBI Gene Expression Omnibus (GEO) database under accession number GSE81365.
