# Supplementary material for: Analysis of Light- and Carbon-Specific Transcriptomes Implicates a Class of G-Protein-Coupled Receptors in Cellulose Sensing
Source: mSphere. 2017 May 10;2(3):e00089-17. doi: 10.1128/mSphere.00089-17 (PMC5425790; doi:10.1128/mSphere.00089-17)
Supplement: Text S1 [file sph003172282s1.pdf]

Analysis of light and carbon specific transcriptomes implicates a class of G-protein coupled receptors in cellulose sensing

Eva Stappler[1] Christoph Dattenböck[1+], Doris Tisch[2+], and Monika Schmoll[1*]

Supplemental Text

Figure S1: Regulation of the cbh2 comprising gene cluster **2**

Coregulation of uncharacterized genes with known genes relevant for cellulase expression **3**

Functional category analyses of induction specific genes **4**

Genes specific for cellulase inducing conditions in darkness 4

Genes specific for cellulase inducing conditions in light 4

Genes specific for cellulase inducing conditions independent of light 5

Figure S2. Schematic representation of functional categories with induction specific regulation in darkness. **6**

Figure S3. Schematic representation of functional categories with induction specific regulation in light. **7**

Genes associated with repression of cellulase gene expression **8**

Devoting resources to specific functions in light and darkness **9**

Figure S4. Analysis of growth of selected GPCR deletion strains **10**

REFERENCES **10**

Figure S1

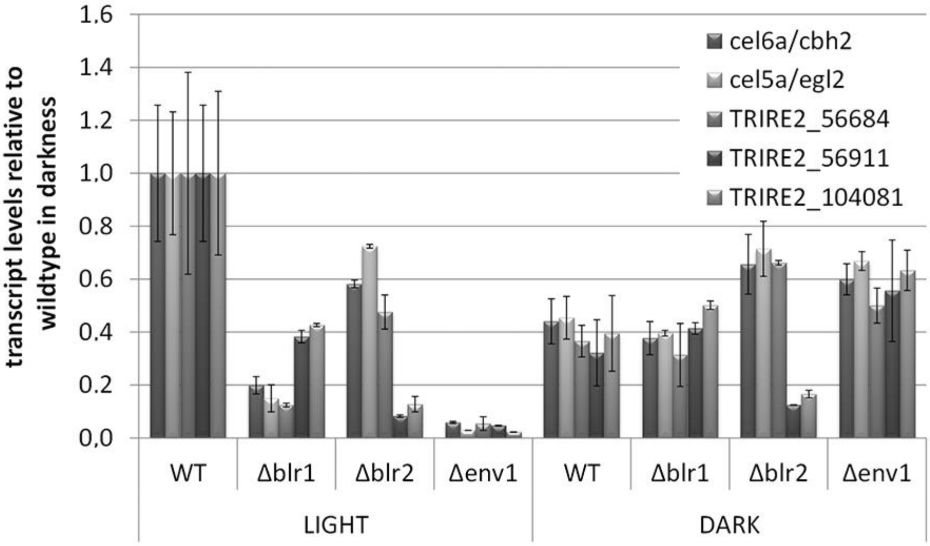

Figure S1: Regulation of the cbh2 comprising gene cluster (Scaffold 3, 12000-94000). Transcript levels in the wildtype and in photoreceptor deletion strains of genes within the cluster (*cel6a/cbh2, cel5a/egl2,* one predicted sugar transporter (TR_56684), one putative urea transporter (TR_56911) and one FMN dependent oxidoreductase (TR_104081)) are shown relative to transcript levels in the wildtype in constant light. Data shown are taken from the dataset published in (1) which describes gene regulation by the photoreceptors BLR1, BLR2 and ENV1 in light and darkness upon growth on cellulose. Errorbars reflect standard deviations between biological replicates ((1); GenBank Accession number GSE36448).

Coregulation of uncharacterized genes with known genes relevant for cellulase expression

Genes exerting similar functions often show comparable regulation patterns across different growth conditions (2-4). Therefore we analyzed coregulation of genes across all carbon sources studied in light and darkness for the cellulase transcription factor genes *xyr1*, *ace2*, *ace3* (activators) as well as *cre1* and *ace1* (repressors) (5, 6). Coregulated genes were narrowed down by increasing the minimum similarity level in the Hierarchical Cluster Explorer (HCE3.5) to 250 genes for *xyr1*, which included *ace1*, to 638 genes for *cre1*, which included *ace2* and to 176 genes for *ace3* (Supplementary file 6). The major cellulases *cel6a/cbh2* and *cel7a/cbh1* clustered with *ace3* and also *creB* as well as 6 genes involved in glycosylation, 3 proteases and 6 genes involved in secretion were in this group. For *cre1* and *ace2* we found coregulation with 20 CAZyme encoding genes including *axe1*, *nag1* and *xyn4*, 4 genes involved in glycosylation as well as 22 protein kinases, but interestingly also 10 genes involved in chromatin and histone modification and 10 genes relevant for genome integrity. This finding is in agreement with cellulases being subject to epigenetic regulation and with the role of CRE1 in this process (7, 8). For *xyr1* and *ace1*, coregulation was observed with 20 CAZyme genes including *cip2*, *bgl1*, *cel3c* and *egl5* as well as with the regulator genes *hap5* and *lae1*.

In order to gain more information on yet uncharacterized transcription factors that might be relevant for cellulase gene expression in *T. reesei*, we used the recent annotation by (9) to check for the transcription factor genes coregulated with *ace1*, *ace2*, *ace3*, *xyr1* and *cre1* (Supplementary file 6). In total, 35 additional transcription factors, most of them not yet characterized, were identified as coregulated and might represent promising targets for strain improvement.

Functional category analyses of induction specific genes

Functional categories of gene groups were determined using the online tool at MIPS (http://mips.helmholtz-muenchen.de/funcatDB/) for *Trichoderma reesei*. A p-value of 5.0e-03 was set as threshold for significant enrichment of a functional category in a given gene set. In the following we provide an overview on the enriched gene sets with induction specific genes in darkness, in light and in the overlap between light and darkness. The detailed data for functional category analyses data of genes only up-/down-regulated under inducing conditions in darkness and those only up-/down-regulated under inducing conditions in light are provided in supplementary file 7.

Genes specific for cellulase inducing conditions in darkness

As expected the gene set upregulated under inducing conditions in darkness (see also Figure S1A) shows significant enrichment in polysaccharide metabolism (p-value 5.11 e-06) and interestingly also in genes involved in nutrient starvation response (p-value 1.33 e-04).
Genes downregulated under inducing conditions in darkness (see also Figure S1B) are significantly enriched in functions in amino acid metabolism (p-value 1.38 e-25), C-compound and carbohydrate metabolism (p-value 1.83e-04) and -transport (p-value 7.42 e-06), especially C-1 compound metabolism, but not polysaccharide metabolism. Additionally, also genes involved in secondary metabolism (p-value 1.12 e-06) and energy related functions (p-value 2.32 e-04), specifically the TCA pathway, rRNA synthesis and processing (p-values <e-05) are downregulated under inducing conditions in darkness. Also genes involved in protein synthesis (p-value 3.83 e-09) and ribosome biogenesis (p-value 1.13 e-06) are enriched in this gene set.

Genes specific for cellulase inducing conditions in light

Genes upregulated upon inducing conditions in light (see also Figure S2A) were enriched in functions related to metabolism (p-value 2.58 e-03), particularly C-compound and carbohydrate metabolism (p-value 4.41e-04) as well as transport and polysaccharide metabolism (p-value 1.23 e-05). Interestingly, genes involved in secondary metabolism are enriched in this gene set (p-value 2.12 e- 03) although this function is enriched among genes downregulated under these conditions in darkness. The enrichment of genes associated with autoproteolytic processing (p-value 3.43 e-05) is likely to reflect a reaction to stress conditions. Also perception of nutrients and nutritional adaptation are enriched in this gene set (p-value 2.96 e-03).

In the gene set (see also Figure S2B) with decreased transcript levels in light, significant enrichment was detected for functions in amino acid metabolism (p-value 1.33e.08), energy (p-value 1.45 e-03), respiration (p-value 1.37 e-03), transcription (p-value 3.12 e-03), rRNA synthesis (p-value 9.14 e-09), RNA processing (p-value 1.28 e-08), particularly rRNA processing (p-value 6.70 e-17), protein synthesis (p-value 3.09 e-21) and particularly ribosome biogenesis (p-value 1.49 e-23), ribosomal proteins (p-value 1.29 e-15) and translation (p-value 1.05 e-06).
Hence, the functions in this gene set resemble those for downregulation under inducing conditions in darkness, but in many cases enrichment is even stronger in light. Consequently,

gene regulation is altered for different genes with the same function in addition to adjustment of mechanisms needed specifically in light or darkness.

Genes specific for cellulase inducing conditions independent of light

Functions significantly enriched for genes downregulated under inducing conditions in light and darkness include metabolism (p-value 3.3 e-04), particularly amino acid metabolism (p-value 1.17e-08), energy (p-value 3.29e-03), respiration (p-value 3.40e-03), rRNA synthesis and processing (p-values of e-08), RNA processing (p-value 1.43 e-03), protein synthesis (p-value 1.19e-06), especially ribosome biogenesis (p-value 2.52 e-08) and ribosomal proteins (p-value 5.54e-05), cellular transport (p-value 3.17e-04) including C-compound and carbohydrate transport (p-value 1.45 e-03) and amino acid transport (p-value 8.64 e-05).
The gene set upregulated under inducing conditions in light and darkness is significantly enriched in functions of C-compound and carbohydrate metabolism (p-value 6.07e-04) including sugar, polyol and carboxylate metabolism, and particularly in polysaccharide metabolism (p-value 9.54e-08), moreover nutrient starvation response (p-value 1.17e-04).

Figure S2. Schematic representation of functional categories with induction specific regulation in darkness.

A

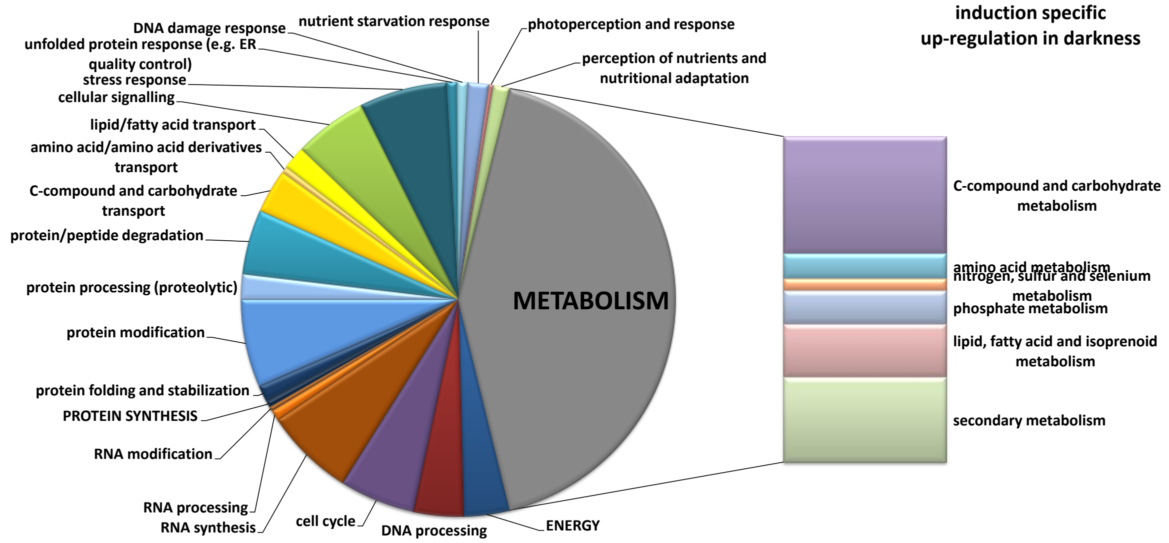

B

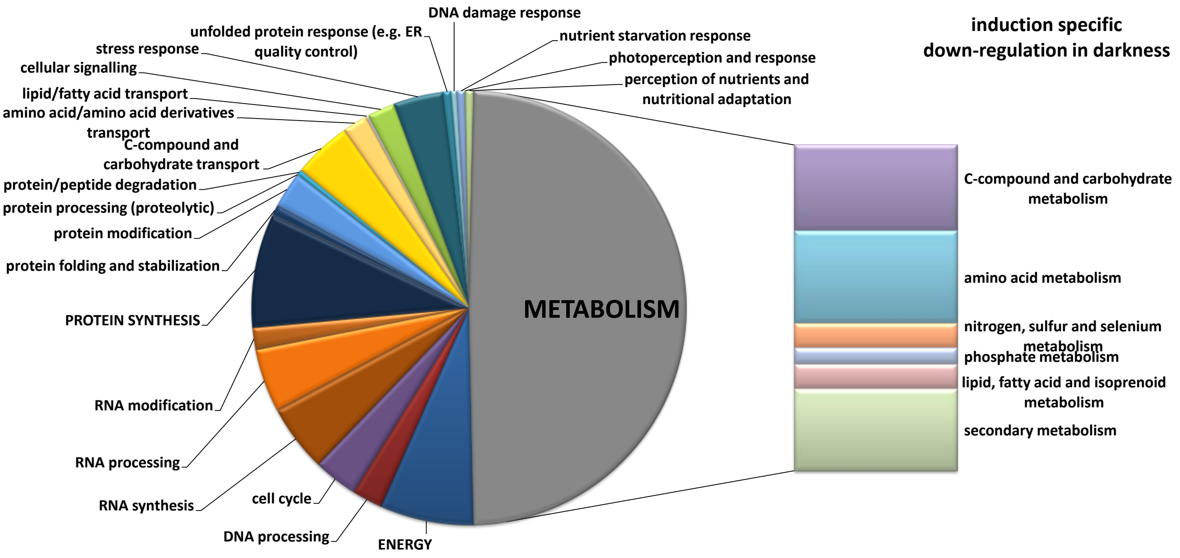

Figure S2. Schematic representation of functional categories with induction specific regulation in darkness. (A) Numbers of genes in selected functional categories that show induction specific up-regulation in darkness. (B) Numbers of genes in selected functional categories that show induction specific down-regulation in darkness.

Significantly enriched functional categories represent a subset of these categories (see supplementary file 7) and are not highlighted here.

Figure S3. Schematic representation of functional categories with induction specific regulation in light.

A

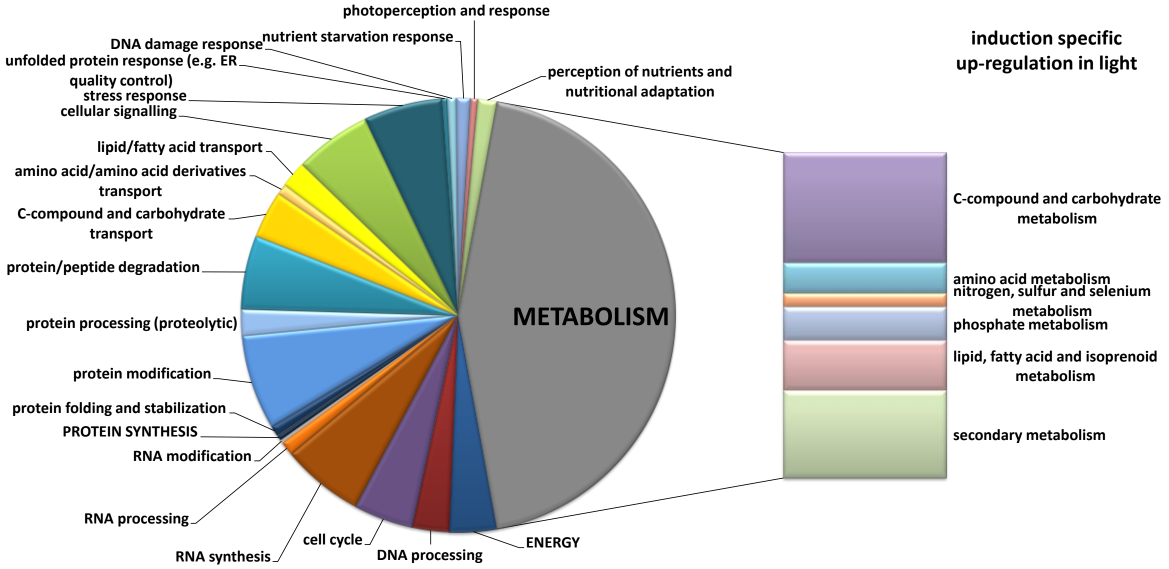

B

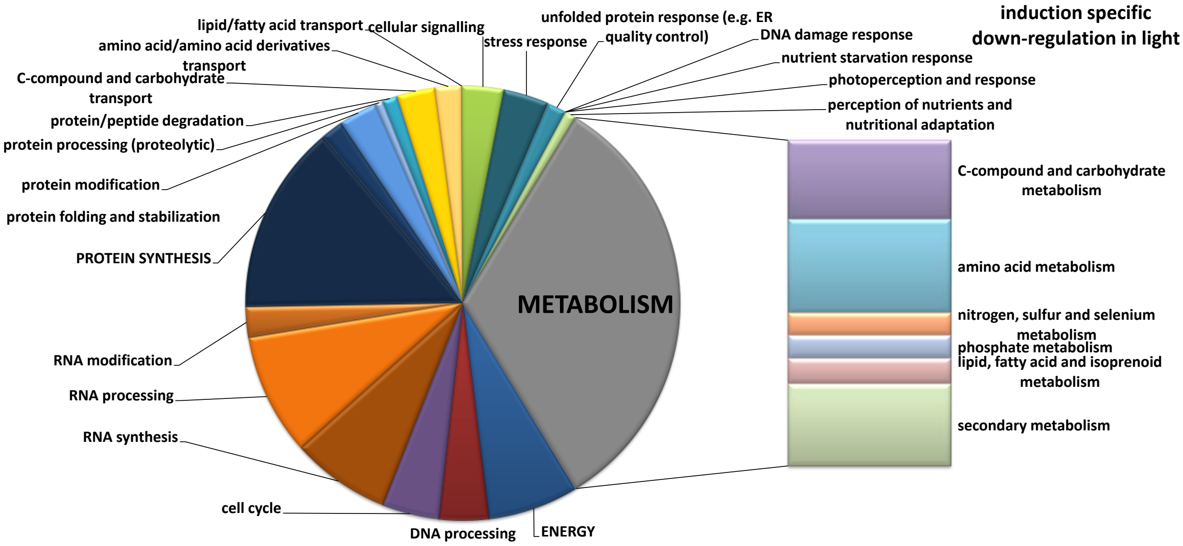

Figure S3. Schematic representation of functional categories with induction specific regulation in light. (A) Numbers of genes in selected functional categories that show induction specific up-regulation in light. (B) Numbers of genes in selected functional categories that show induction specific down-regulation in light.

Significantly enriched functional categories represent a subset of these categories (see supplementary file 7) and are not highlighted here.

Genes associated with repression of cellulase gene expression

Upon growth on glucose, induction of cellulase gene expression does not occur, even in the presence of an inducer (10). In contrast, addition of inducing compounds to mycelia grown on glycerol does result in cellulase gene expression. Hence comparison of the transcriptomes on glucose and glycerol allows us to gain insight into which processes are involved in repression. We found 570 genes to be differentially regulated between glucose and glycerol (Supplementary file 9). Genes consistently regulated in light and darkness under these conditions are considered most relevant for repression by glucose. The 60 genes downregulated upon growth on glucose versus glycerol in light and darkness are enriched in functions of metabolism (p-value 1.67e-11) and especially amino acid metabolism (p-value 9.44 e-07) as well as C-compound and carbohydrate metabolism (p-value 7.54 e-08) and lipid, fatty acid and isoprenoid metabolism (p-value 1.44 e-05), but also secondary metabolism (p-value 1.86e-04). Additionally, energy related functions (p-value 1.98e-05) including the TCA cycle (p-value 3.37e-04) and fermentation (p-value 1.25e-03) are enriched in this gene group.

These genes include two Zn binuclear cluster domain transcription factors (TR_72611 and TR_121107 with more than 8fold downregulation) as well as six transporters, most of them predicted sugar transporters (TR_50618, TR_68812, TR_62502 and TR76800, all with more than 9fold regulation), which supports the hypothesis of inducer exclusion occurring in the presence of glucose (11).

The 25 genes upregulated on glucose versus glycerol in light and darkness show enrichment in cellular transport functions (p-value 4.33 e-03), including C-compound and carbohydrate transport (p-value 1.98e-03). Among these genes are several strongly regulated transporters including a putative sodium/glucose cotransporter (TR_57015) and a predicted galactose permease (TR_106556).

Devoting resources to specific functions in light and darkness

Due to the considerable number of genes regulated in light and darkness, we wanted to evaluate between which functions the majority of resources in terms of transcript abundance are shifted in light and darkness. We therefore analyzed the functions of the 100 most strongly regulated genes in terms of absolute signal strength change on the different carbon sources for cellulose, sophorose and glucose (Supplementary file 8).

On cellulose the positive shift of abundance in light compared to darkness of those 100 genes comprised 37.5 % of the total signal of positive changes. Significant enrichment among upregulated genes was only observed for functions in metabolism (p-value 3.20 e-04), particularly sulfur metabolism and assimilation (p-values e-05), but also C-compound and carbohydrate metabolism (p-value 9.84 e-04) and especially polysaccharide metabolism (p-value 9.54 e-07). Among the downregulated genes no significant enrichment was observed.

Analysis of the genes in this group revealed that considerable resources are devoted to increase transcription of CAZyme genes in light, with 13 glycoside hydrolases including *cel6a* and *cel7a*, the major cellulase genes as well as *egl1, egl2, bxl1, cel1b* and *abf3*. Additionally the carbohydrate esterases *aes2* and *axe1* as well as two CBM family protein encoding genes including *cip1* are among the genes most strongly increasing in abundance in light. Moreover, the protease encoding gene TR_77579, ten genes involved in sulfur metabolism, 4 transcription factors and 4 transporters including TR_3405 are in this group. Surprisingly, also 5 genes involved in sexual development were found, although the fungus had been cultivated in liquid culture for this study. From the genes related to light response, *frq1, env1* and *vel1* were in this gene group as well as the G-protein coupled receptor TR_121990 (PTH11like).

Genes with strongest decrease in transcript levels (100 genes with 27.3 % of total decreased signals) included 5 glycoside hydrolases potentially related to cell wall modification including *chi18-18*, two proteases (TR_121306 and TR_119876), two genes involved in secretion (TR_121251 and TR_23263), a gene related to *N. crassa vib-1* and 4 transporters.

Next, we wanted to compare these data from continued growth on cellulose to resource allocation early after cellulase induction as reflected by growth on sophorose in light versus darkness. There, peptide transport (p-value 1.48 e-03), cellular export and secretion (p-value 2.10 e-03) and transmembrane signal transduction (p-value 3.74e-04) are enriched among strongly upregulated functions. However, sulfur metabolism and assimilation (p-values e-04), C-compound and carbohydrate metabolism (p-value 2.61 e-03) and particularly polysaccharide metabolism (p-value 4.35 e-05) are enriched among strongly downregulated functions, which contrasts with cellulose.

On glucose it can be expected that resources are allocated in a different way than on the inducing carbon sources, because initiation and maintenance of enzyme biosynthesis is not required. Interestingly, the assignment of resources was different on glucose between light and darkness. The 100 most strongly upregulated genes on glucose were enriched in rRNA processing (p-value 3.16e-05), protein synthesis (p-value 4.87e-35), especially with respect to ribosomal proteins (p-value 5.18e-34) and translation (p-value 9.04e-30). Also genes encoding GTP binding proteins (8.01e-06) and others with binding function or cofactor requirement (p-value 6.23e-10) were enriched in this gene group. Metabolic genes were clearly underrepresented.

Among genes most strongly downregulated in light compared to darkness on glucose, genes with functions in metabolism (p-value 3.28e-03) and especially amino acid metabolism (p-value 1.35e-08), but also nitrogen, sulfur and selenium metabolism (p-value 8.37e-04) as well as C-compound and carbohydrate transport (p-value 3.34e-04) were significantly enriched.

Figure S4. Analysis of growth of selected GPCR deletion strains

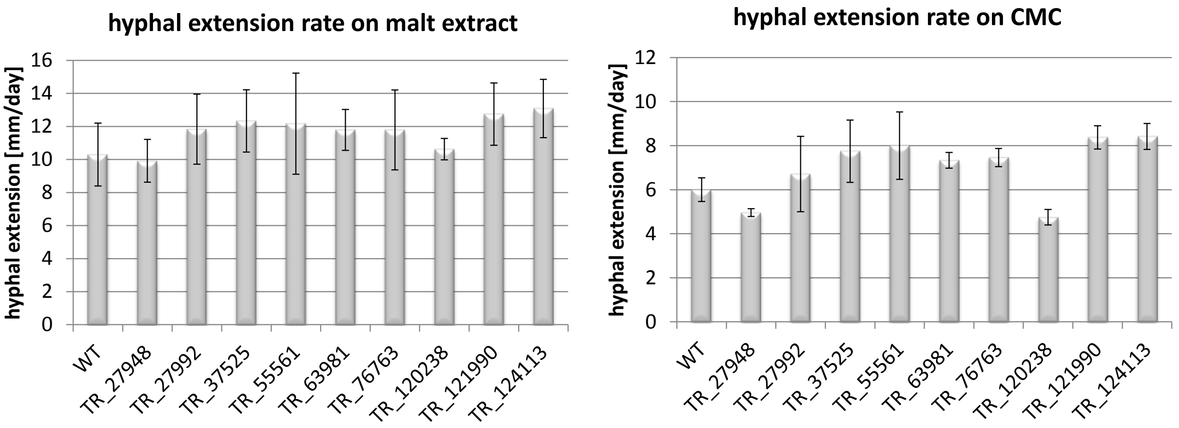

Figure S4. **Analysis of growth of selected GPCR deletion strains**
Strains were grown on malt extract agar (3 % w/v) or carboxymethylcellulose (CMC, 1 % w/w) and hyphal extension was recorded daily. At least three biological replicates per gene were considered. Errorbars show standard deviations.

REFERENCES

1. **Tisch D, Schmoll M.** 2013. Targets of light signalling in *Trichoderma reesei*. BMC Genomics **14:**657.
2. **Eisen MB, Spellman PT, Brown PO, Botstein D.** 1998. Cluster analysis and display of genome-wide expression patterns. Proc Natl Acad Sci U S A **95:**14863-14868.
3. **Harmer SL, Hogenesch JB, Straume M, Chang HS, Han B, Zhu T, Wang X, Kreps JA, Kay SA.** 2000. Orchestrated transcription of key pathways in *Arabidopsis* by the circadian clock. Science **290:**2110-2113.
4. **Kasuga T, Townsend JP, Tian C, Gilbert LB, Mannhaupt G, Taylor JW, Glass NL.** 2005. Long-oligomer microarray profiling in *Neurospora crassa* reveals the transcriptional program underlying biochemical and physiological events of conidial germination. Nucleic Acids Res **33:**6469-6485.
5. **Hakkinen M, Valkonen MJ, Westerholm-Parvinen A, Aro N, Arvas M, Vitikainen M, Penttila M, Saloheimo M, Pakula TM.** 2014. Screening of candidate regulators for cellulase and hemicellulase production in *Trichoderma reesei* and identification of a factor essential for cellulase production. Biotechnol Biofuels **7:**14.
6. **Schmoll M, Seiboth B, Druzhinina I, Kubicek CP.** 2014. Genomics Analysis of Biocontrol Species and Industrial Enzyme Producers from the Genus *Trichoderma* p233-264. *In* Nowrousian M (ed), Fungal Genomics, 2 ed, vol 13. Springer, Berlin, Heidelberg.
7. **Zeilinger S, Schmoll M, Pail M, Mach RL, Kubicek CP.** 2003. Nucleosome transactions on the *Hypocrea jecorina* (*Trichoderma reesei*) cellulase promoter *cbh2* associated with cellulase induction. Mol Genet Genomics **270:**46-55.
8. **Mello-de-Sousa TM, Rassinger A, Pucher ME, dos Santos Castro L, Persinoti GF, Silva-Rocha R, Pocas-Fonseca MJ, Mach RL, Nascimento Silva R, Mach-Aigner AR.** 2015. The impact of chromatin remodelling on cellulase expression in *Trichoderma reesei*. BMC Genomics **16:**588.
9. **Schmoll M, Dattenbock C, Carreras-Villasenor N, Mendoza-Mendoza A, Tisch D, Aleman MI, Baker SE, Brown C, Cervantes-Badillo MG, Cetz-Chel J,**

Cristobal-Mondragon GR, Delaye L, Esquivel-Naranjo EU, Frischmann A, Gallardo-Negrete Jde J, Garcia-Esquivel M, Gomez-Rodriguez EY, Greenwood DR, Hernandez-Onate M, Kruszewska JS, Lawry R, Mora-Montes HM, Munoz-Centeno T, Nieto-Jacobo MF, Nogueira Lopez G, Olmedo-Monfil V, Osorio-Concepcion M, Pilsyk S, Pomraning KR, Rodriguez-Iglesias A, Rosales-Saavedra MT, Sanchez-Arreguin JA, Seidl-Seiboth V, Stewart A, Uresti-Rivera EE, Wang CL, Wang TF, Zeilinger S, Casas-Flores S, Herrera-Estrella A. 2016. The Genomes of Three Uneven Siblings: Footprints of the Lifestyles of Three *Trichoderma* Species. Microbiol Mol Biol Rev **80:**205-327.

10. **Ilmen M, Saloheimo A, Onnela ML, Penttila ME.** 1997. Regulation of cellulase gene expression in the filamentous fungus *Trichoderma reesei*. Appl Environ Microbiol **63:**1298-1306.
11. **Kubicek CP, Messner R, Gruber F, Mandels M, Kubicek-Pranz EM.** 1993. Triggering of cellulase biosynthesis by cellulose in *Trichoderma reesei*. Involvement of a constitutive, sophorose-inducible, glucose-inhibited beta-diglucoside permease. J Biol Chem **268:**19364-19368.
